# Earlier and prolonged respiratory syncytial virus (RSV) seasons in young children compared to adults: implications for prevention in infants

**DOI:** 10.1128/spectrum.01599-25

**Published:** 2025-09-25

**Authors:** Yuan Chao Xue, Natalie Williams-Bouyer, Ping Ren, Janak A. Patel

**Affiliations:** 1 Department of Pathology, University of Texas Medical Branch 198642, Galveston, Texas, USA; 2 Department of Infection Control & Healthcare Epidemiology, University of Texas Medical Branch 12338, Galveston, Texas, USA; 3 Department of Pediatrics, University of Texas Medical Branch 12338, Galveston, Texas, USA; End TB Dx Consulting LLC, San Diego, California, USA

**Keywords:** RSV seasonality, pediatrics, epidemiology

## Abstract

Respiratory syncytial virus (RSV) poses a major health concern, particularly for young children and older adults. In this 10-year, single-center retrospective study (15 May 2015 to 14 May 2025), we analyzed RSV positivity rates to characterize local epidemiologic trends. When stratified by age, children ≤2 years consistently exhibited the highest positivity rates. Using the Centers for Disease Control and Prevention’s 3% positivity threshold, we found that the RSV season in the general population in our region began in early July and ended in late December, approximately 3 months earlier than the national average. Among infants ≤2 years, the season lasted 5 weeks longer on average than in the general population. These findings highlight regional variability in RSV seasonality and the earlier onset and extended duration of RSV activity in young children. This age group appeared particularly susceptible and likely played a key role in initiating and sustaining community transmission. Monitoring RSV positivity specifically in children ≤2 years, separate from the general population, may enhance local surveillance accuracy and support more timely decisions regarding RSV immunoprophylaxis and vaccination strategies.

**IMPORTANCE:**

Respiratory syncytial virus (RSV) is a major health concern, especially for young children and older adults. In this study, we explored whether different age groups changed the timing of RSV season. This is important because public health guidelines, hospital preparedness, and preventive strategies like antibody prophylaxis and vaccination rely on accurate RSV season timing. By showing how RSV trends differ by age, our findings can help improve seasonality responses and ensure that preventive measures reach high-risk groups, especially infants, at the right time.

## OBSERVATION

Respiratory syncytial virus (RSV) is a leading cause of lower respiratory tract infections, posing a significant public health threat, particularly to young children and older adults. Among children under 5 years of age, RSV is a primary cause of bronchiolitis and pneumonia, often necessitating hospitalization (1). In older adults and individuals with underlying conditions, RSV infections contribute to substantial morbidity and mortality, further emphasizing its clinical significance (2). Understanding the dynamics of RSV transmission and seasonality is critical to mitigating its healthcare burden, specifically in these high-risk populations (1).

In the United States, the RSV season typically spans from October to April, with regional and local variations (3). The Centers for Disease Control and Prevention (CDC) defines the start and end of the RSV season as the first and last occurrence of two consecutive weeks with positivity rates of ≥3% based on molecular diagnostics among all tested individuals (3). However, this threshold may miss important age-related trends, particularly the disproportionate burden in younger children seen in both outpatient and inpatient settings (4). Monitoring RSV patterns separately in high-risk pediatric populations is crucial to uncover more nuanced patterns and improve the timing of prevention strategies, including passive immunization in infants and vaccination of pregnant women and older adults.

This 10-year, single-center retrospective study aimed to characterize local RSV testing and positivity rates, stratified by age, to better understand RSV seasonality in a regional context.

We extracted de-identified data from the University of Texas Medical Branch (UTMB) Epic electronic medical record system for patients receiving care at UTMB clinics, emergency rooms, urgent care centers, and hospitals. The data set included results, testing dates, patient gender, and age at the time of testing.

Data cleaning (excluding entries missing dates or age) and analysis were performed in RStudio (Version 2024.12.1 + 467), resulting in 92,921 eligible entries. Data consistency and integrity were also reviewed.

For analysis and visualization, we used the following R packages: *ggplot2* (Version 3.5.1) for data visualization, *dplyr* (Version 1.1.4) and *tidyverse* (Version 2.0.0) for data manipulation, *tidyr* (Version 1.3.1) for data tidying, *lubridate* (Version 1.9.4) for date handling, and *zoo* (Version 1.8-12) for time-series analysis. To evaluate seasonal patterns, *t*-tests were used for comparison of group means, and Z-tests with Bonferroni correction were used for time-series analysis. This study was approved by the UTMB Institutional Review Board (#24-0390).

From 15 May 2015 to 14 May 2025, the overall number of RSV tests at UTMB steadily increased, but the distribution of testing across age groups did not change significantly (*P* >0.05), and weekly positivity rates remained relatively stable across each annual cycle (data not shown). To explore age-specific trends, data were stratified into three age groups: ≤2 years, 3–18 years, and ≥19 years.

**Fig 1:**
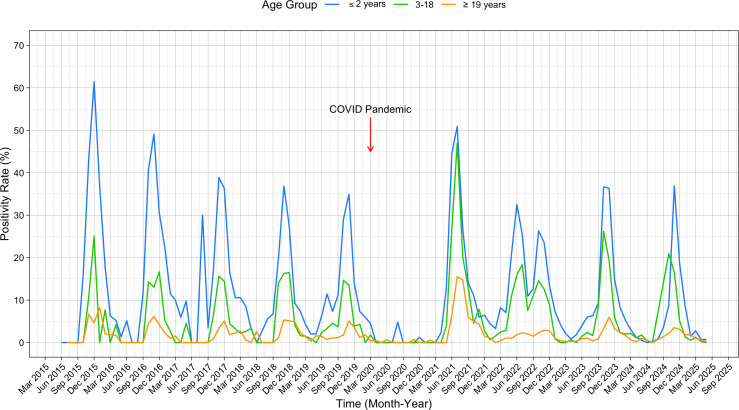
RSV seasonality from 2015 to 2025 at a single-center medical institution. The weekly positivity rate among three different age groups: ≤2 years (blue), 3–18 (green), and ≥19 years (orange).

Adults (≥19 years) accounted for 48.9% of all RSV tests during the study period, totaling 45,468 tests. In comparison, the pediatric population underwent a combined number of 47,453 tests. This included 33,106 tests (35.7%) for children ≤2 years and 14,347 tests (15.4%) for those aged 3–18 years. Despite lower testing volumes in younger age groups, children consistently exhibited higher positivity rates than adults (Fig. 1). Notably, children ≤2 years had significantly higher positivity rates than all other age groups and the overall population (Z-test: *P* <0.001).

According to the CDC definition, an RSV season spans from the first to the last occurrence of two consecutive weeks with positivity rates of ≥3% using molecular testing methods. In our region, RSV seasons typically began in early July and ended in late December, with the exception of atypical patterns observed from 2021 to 2023 due to the COVID-19 pandemic. During these years, a major summer outbreak occurred in 2021, followed by a distinct two-peak season in 2022–2023. Then, the 2023–2024 season reverted to the traditional fall-to-late-winter/early-spring pattern. Importantly, children ≤2 years consistently showed an earlier season onset by 1 week (28 June vs 8 July; *t*-test: *P *= 0.043) and a later end date by 4 weeks (22 January vs 26 December; *t*-test: *P *= 0.0019) compared to the overall population (Table 1). As a result, their RSV season was significantly longer by 5.1 weeks (33.5 weeks vs 28.4 weeks; *t*-test: *P* = 0.024), excluding the atypical 2021–2023 seasons (Table 1). To assess the resilience of RSV seasonality in children ≤2 years, we compared the impact of applying a higher positivity threshold of 5%. Relative to the 3% threshold, the 5% threshold shortened the RSV season by 4.3 weeks in children ≤2 years but by 8.7 weeks in the overall population (Table 1).

**TABLE 1:** RSV seasonality changes in the ≤2 years group and the general population when 3% or 5% positivity thresholds applied

	Average season starting date		Average season ending date		Average duration (weeks)	
Positivity threshold	3%	5%	3%	5%	3%	5%
≤ 2 years	28 June	4 July	22 January	5 January	33.5	29.2
General population	8 July	2 August	26 December	29 November	28.4	19.7

In this study, we confirmed regional variability in RSV seasonality, consistent with data from the CDC’s National Respiratory and Enteric Virus Surveillance System (https://www.cdc.gov/nrevss/php/dashboard/index.html). Notably, the RSV season in our region of southern Texas appeared to be approximately 2 weeks longer than that reported for Region 6, which encompassed 16–21 laboratories’ data from New Mexico, Texas, Oklahoma, Louisiana, and Arkansas (3). Age-stratified analysis revealed that adults accounted for nearly half of the total RSV tests; however, the highest positivity rates were consistently observed in the pediatric population, particularly among children ≤2 years of age. These findings are aligned with those reported by Tran *et al.,* who also showed high positivity rates in children under 4 years (5).

Furthermore, we observed that children ≤2 years consistently experienced an earlier onset and longer duration of RSV seasons compared to the general population, suggesting that they may be more susceptible to RSV infection and at greater risk for severe disease than older children and adults. When the positivity threshold increased from 3% to 5%, the RSV season had less reduction in children ≤2 years than in the general population. This highlights the resiliency of RSV seasonality in young children, even when stricter criteria were applied. In conclusion, our observations indicate that RSV surveillance in children ≤2 years should guide the timing of immunoprophylaxis administration in infants or maternal RSV vaccination, rather than relying solely on data from the general population. However, this study’s single-center design may limit the generalizability of the findings, as they reflect a specific regional experience.
